# Pilot study of a manualised mental health awareness and stigma reduction intervention for Black faith communities in the UK: ON TRAC project

**DOI:** 10.1007/s00127-023-02492-2

**Published:** 2023-05-27

**Authors:** Louisa Codjoe, Joelyn N’Danga-Koroma, Claire Henderson, Heidi Lempp, Graham Thornicroft

**Affiliations:** 1 Health Service and Population Research Department, Institute of Psychiatry, Psychology and Neuroscience (IoPPN), King’s College London, De Crespigny Park, London, SE5 8AF UK; 2 Department of Inflammation Biology, Centre for Rheumatic Diseases, School of Immunology and Microbial Sciences, King’s College London, Weston Education, 10, Cutcombe Rd, London, SE5 9RJ UK; 3 Centre for Implementation Science and Centre for Global Mental Health, Institute of Psychiatry, Psychology and Neuroscience, King’s College London, De Crespigny Park, London, SE5 8AF UK

**Keywords:** Mental health awareness, Stigma reduction, Black faith

## Abstract

**Background:**

Building partnerships between mental health services and Black faith communities to co-produce culturally tailored interventions is an essential step towards improving access to services and reducing stigma among the Black population. Given that Black faith organisations are considered a primary source of emotional and psychological support they are well positioned as ‘gatekeepers’ for services, to overcome barriers to engagement and build trusting relationships with the Black community. The aim of this paper is to pilot a manualised mental health awareness and stigma reduction intervention for Black faith communities in the UK, and to make an initial assessment of feasibility, acceptability and outcomes.

**Methods:**

This study employed a mixed methods pre–post-design, based upon the Medical Research Council Framework (MRC) for complex interventions, and the Implementation Science Research Development.

**Results:**

The qualitative assessments indicate that the intervention was found overall to be acceptable and feasible to the Black faith community population. This pilot study did not find statistically significant changes for the Mental Health Knowledge schedule (MAKS), Reported and Intended Behaviour Scale (RIBS), intended help-seeking or willingness to disclose (Attitudes to Mental Illness Survey) measures. However, the direction of all the non-significant changes in these measures suggests positive changes in mental health knowledge, a reduction in participants’ desire for social distance, and greater willingness to disclose personal experiences of mental health problems. A statistically significant improvement in the Community Attitudes towards Mental Illness (CAMI) scale results indicated a lower level of stigmatising attitudes towards people with lived experience of mental health conditions (PWLE), and an increase in tolerance and support towards PWLE after the intervention. Significant improvement in the willingness to disclose measure suggests increased preparedness to seek help amongst participants, a lesser desire for social distance, and greater willingness to engage with PWLE after the intervention. Three key themes, including 9 subthemes were identified from the qualitative data analysis: (i) initial implementation and intention to adopt; (ii) perceived suitability and usefulness of intervention to address cultural issues relating to mental health in the Black community; and (iii) strengthening the capacity of faith leaders.

**Conclusions:**

This ON TRAC pilot study shows that the intervention was feasible and acceptable, and that it has promising positive impacts and next requires larger scale evaluation. These results demonstrate that the intervention was a culturally acceptable way to potentially increase mental health awareness and reduce stigma in Black faith communities.

**Trial registration:**

ISRCTN12253092.

## Background

The need to improve service access, experience, and outcomes for members of Black African and Caribbean groups in the UK has been identified as a key priority in national mental health policies for many years. Yet, people in these groups continue to experience poorer treatment and worse recovery outcomes in comparison to other ethnic groups (Mantovani et al. [18]). Barnett et al. [2] found that Black people are 2.3–2.5 times more likely than White people to contact mental health services at more severe stages of mental ill health and experience the more restrictive elements of mental health services in the UK. Members of Black communities are reluctant to access mental health services at earlier stages due to negative perceptions of services and fear of discriminatory treatment by professionals [22].

Indeed, Keating et al. [16] found fear and mistrust of mental health services among the UK Black community were associated with a perception that mental health services reinforce the structural position of Black people, in relation to racism and discrimination experienced in wider society. Research exploring perceptions of mental health and relevant services among the UK Black population found that cultural beliefs and understandings of social and emotional difficulties, coping mechanisms and healing practices may reinforce negative stigmatising attitudes around mental health and illness, and prevent people from accessing timely help from mental health services (Mantovani et al. [18]). In a multi-cultural society, the reported lack of adequate training and service provision about the impact of racism, discrimination and widespread ethnic inequalities presents a politically, socially, and structurally complex situation (Banaji et al. [1]). Cenat [4] suggested that health care providers need to go beyond ensuring that their offer is non-racist, but instead work towards being anti-racist. This includes the development of interventions that address the real needs of the Black community and suggests that ‘the role of culturally appropriate interventions and conversations about ‘*race, ethnicity, religion, spirituality and culture help build a strong therapeutic alliance*’ (Cenat [4]: p930).

Building partnerships between mental health services and Black faith communities to co-produce culturally tailored interventions is an essential step towards improving access to services and reducing stigma among the Black population [7]. Given that Black faith organisations are considered a primary source of emotional and psychological support (Codjoe et al. [5]), they are well positioned as ‘gatekeepers’ to services, to overcome barriers to engagement and build trusting relationships with the Black community [16]. Joint working provides opportunities for mental health services to improve their understanding of cultural practices and beliefs relating to mental health and illness, while working to raise awareness of mental health and services within the wider Black community. However, we have found no research on mental health awareness or stigma interventions for members of Black faith communities in the UK.

This pilot study reports on the initial delivery of an in-person mental health awareness intervention, specifically for members of Black faith communities in South London.

The overall aim of this study was to assess the feasibility of the in-person delivery version of the ON TRAC intervention, which is a mental health awareness intervention for members of Black faith communities. The specific study objectives were to assess the feasibility and acceptability of

(i) Recruiting participants to take part in the study.
(ii) Delivering the in-person intervention
(iii) Conducting quantitative and qualitative assessments
(iv) Conducting a post group qualitative semi-structured focus group.

The qualitative and quantitative findings suggest that recruitment to the ON TRAC study, delivery of the intervention, conducting quantitative and qualitative assessments and a post group qualitative semi-structured focus group feasible and acceptable to this population.

## Methods

### Design

This study employed a mixed methods pre–post-design, based on the Medical Research Council Framework (MRC) for complex interventions (MRC [19]), and the Implementation Science Research Development (ImPres) tool (ImPres [17]). In addition, findings from a systematic review examining the available evidence for interventions to promote mental health and reduce stigma in Black faith communities Codjoe et al. [7] identified a number of factors or ‘active ingredients’ that contribute to the effectiveness of the interventions reviewed. As a very under researched area, the information available was limited. These factors were incorporated into the study design and the intervention. Specifically, the study design should include standardised measures particularly of stigma and use a combination of qualitative and quantitative methods. With respect to the intervention, Codjoe et al. [7] noted the importance of creating a manualised intervention, and ensuring that implementation and content of the intervention addressed the fear, denial, stigma and mistrust in mental health services. Within the review, a framework was adapted from Coleman et al. [8] and identified enablers and mediators which contribute to an effective intervention. These were also included within the study and intervention design. For example, enablers included ensuring a balance between faith and health beliefs, effective engagement with faith communities and ensuring leadership support. Regarding mediators, the intervention content included culturally relevant frameworks, promoted the development of faith group mental health champions, the need for flexible times to hold the group and a focus on developing meaningful, collaborative dynamics. Findings indicated that prospective Black faith group participants wanted to have the tools to be able do confidently broach the subject of mental health, understand how to identify common mental health difficulties and where to signpost. Understandably, there was also a recognised need for conversation around how faith and spirituality can coexist with mental health. The review [7] reported that it was important to consider the ethnicity of staff delivering the group and ensure their ability to be able to empathise and connect with the lived experience of participants.

Baseline data were gathered in January 2020 and the follow up time point was at 1 week post-intervention. Due to the challenges associated with the COVID-19 pandemic, there were some delays in the collection of follow-up data. Most data were collected between April and May 2020 with all data collection completed by September 2020.

A qualitative component was included to explore participants’ views on the acceptability, appropriateness, and potential benefits of the intervention. We conducted semi-structured face-to-face interviews with a number of participants and face-to-face focus group participants a week after completion of the course. These data from these qualitative assessments will inform the further development and implementation of the ON TRAC intervention.

## Measures

The following outcome measures were used to compare differences in participants’ mental health awareness, knowledge, attitudes, and willingness to disclose mental health difficulties, both at baseline and post-intervention.

Mental health-related knowledge was measured by the Mental Health Knowledge Schedule (MAKS) [10]. The MAKS comprises two parts, Part A which includes six items covering stigma-related mental health knowledge areas: help seeking, recognition, support, employment, treatment, and recovery, and Part B which includes six items that inquire about classification of various conditions as mental illnesses. Overall test–retest reliability of the MAKS is 0.71 (Lin’s concordance statistic) and the overall internal consistency among items 1–6 is *α* = 0.65.

Attitudes towards mental illness were measured using the 26-item questionnaire based on the 40-item Community Attitudes toward the Mentally Ill (CAMI) scale [23], with an added item on employment-related attitudes. Items referred to attitudes about social exclusion, benevolence, tolerance, and support for community mental healthcare and were rated from 1 (strong disagreement) to 5 (strong agreement). The overall internal consistency for these CAMI items has been reported as *α* = 0.87 (Evans-Lacko et al. [12]). The CAMI-prejudice and exclusion subscale (CAMI-PE) and the tolerance and support (CAMI-TS) subscales were included in the analysis.

Desire for social distance was measured by the four-item intended behaviour (IB) subscale of the Reported and Intended Behaviour Scale (RIBS) [11]. This assesses the level of desired future contact and behaviour towards people with mental health problems, in terms of four different contexts: living with, working with, living nearby, and continuing a relationship with someone. RIBS IB is scored so that a higher score indicates more positive behaviour: less desire for social distance, and the total score was standardised. The overall test–retest reliability of total RIBS score is 0.75 (Lin’s concordance statistic) and the overall internal consistency among items is *α* = 0.85.

Intended help seeking and willingness to disclose were measured using single item questions adapted from the Attitudes to Mental Illness Survey [13]. These items focus on willingness to seek help from a GP and disclose mental health difficulties to family and friends and to employers.

### Setting

This study took place within two local government (council) borough in South London. These areas are very diverse, both culturally and ethnically. For example, both have approximately (60%) white groups making them significantly more ethnically diverse than the UK as a whole (88%) (Statistics taken from https://urbanhealth.org.uk/insights/reports/futures-scenarios-for-lambeth-and-southwark) [accessed 30 August 2022]. These areas also have large numbers of Black Majority Churches (BMC) with over 240 such churches in one of the boroughs [21].

## Eligibility criteria

### Inclusion criteria

Black Majority Church (BMC) participants: aged over 18 years who are members of a Black majority, or a Black-led Church based in South London. Participants who did not self-identify as Black were permitted to engage in the intervention, on the basis that they attended or were affiliated with a Black Majority or Black Led church. Participants needed to have an adequate understanding of English to participate in the planned intervention and the study assessments, and to provide valid written, informed consent.

### Exclusion criteria

Participants who do not give written, informed consent.

### Recruitment

Mental health awareness community events led by the local National Health Service (NHS) Mental Health Trust (service provider organisation) were used to promote the study and invite people to take part. Study flyers were distributed to local Black majority and Black-led churches in South London in person and via email. The study was advertised by sending email flyers through networks within the local NHS Trust and local communities. Individuals who expressed an interest in taking part in the study were screened for eligibility by the research team. Potential participants were encouraged to register for the pilot study two months before the intervention start date. People who met the inclusion criteria were provided with a Study Information Sheet and given the opportunity to ask questions about the project, before written informed consent was invited.

For pilot studies, Julious [14] recommends an appropriate sample size of 12, to test the initial feasibility of novel interventions, or for studies that may involve population groups who may have not been previously investigated in research. The target sample size for this study was, therefore, *n* = 20. This allowed for sample attrition.

### Intervention

The ON TRAC intervention consists of a 10-session, mental health awareness and stigma reduction training course for members of Black faith communities. The course is designed to be engaging and educational, providing participants with opportunities to improve their knowledge around mental health and mental illness, whilst developing skills in communication and active listening. The course is intended to support Black faith community members to improve their skills in supporting members of their congregation, and their families, who may be experiencing mental health difficulties.

### Intervention development

The ON TRAC intervention was co-designed in collaboration and co-produced with members of local Black faith communities, NHS mental health professionals, the ON TRAC research team and the ON TRAC Advisory Group which consisted of professionals and members of local Black faith communities. The design and development of the intervention consisted of the following phases.

### Phase 1—scoping project

The initial phase consisted of a scoping project, and was conducted by the lead researcher (LC), a local Black pastor and NHS mental health professionals. In the scoping project, the team engaged with 85 members of Black Majority Churches and Fellowship groups in South London. This included conducting focus groups and semi-structured interviews with the participants, to explore the feasibility and acceptability of the concept of ON TRAC mental health awareness training course (Codjoe et al. [6]).

### Phase 2—development of a manualised mental health awareness training course

**Fig. 1:**
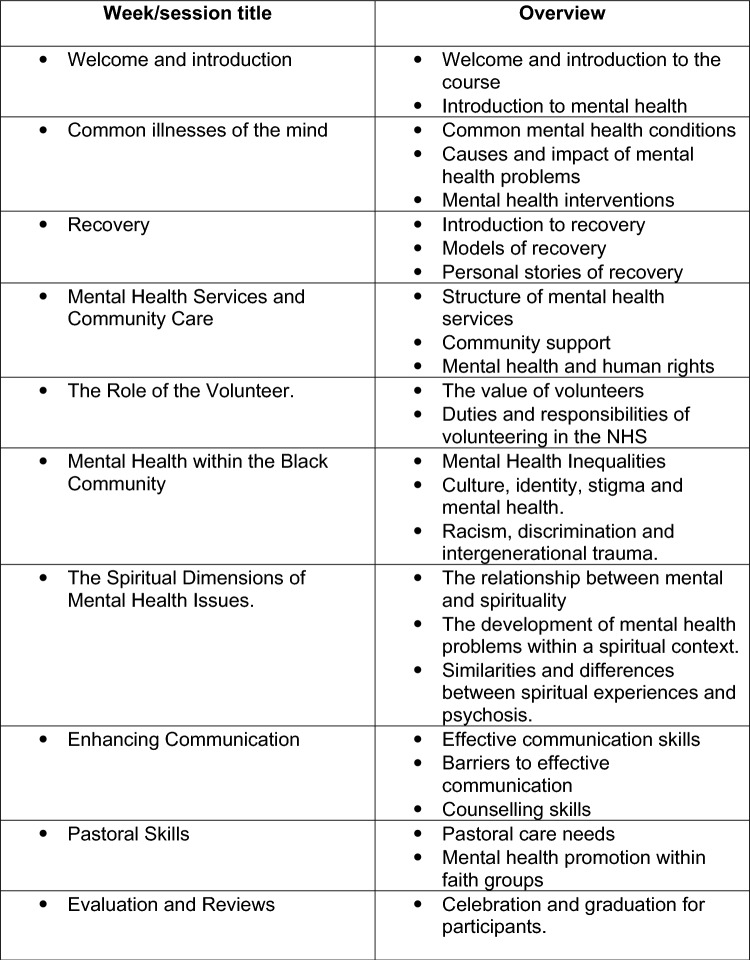
Summary of session content

In June 2019, members of the ON TRAC research team met with the Steering Group to identify the main content to include in the ON TRAC manual. The steering group (*n* = 8) consisted of leaders from Black faith communities, staff from the mental health trust and ON TRAC project. This ensured that the content was culturally appropriate and acceptable to members of Black faith communities. Following meetings of the Steering Group, members of the research team regularly met with NHS staff to develop the ON TRAC training course manual, consisting of 10 modules related to mental health and Black faith communities (see Fig. 1).

### Intervention delivery

Over 10 weeks, participants took part in a series of 2 h face-to-face sessions, once a week on a weekday evening. The first nine sessions of the course took place in person, at a community centre in South London. The final session was delivered online, as a result of COVID-19 social contact restrictions.

Sessions covered the following topics: mental health, mental illness and recovery; mental health services and community care; the role of the volunteer, mental health within the Black community, the spiritual dimensions of mental health issues, and enhancing communication and pastoral skills. In addition, several sessions focused on developing skills in active listening, communication, and pastoral skills for supporting congregation members who may be experiencing mental health difficulties (see Fig. 1).

Throughout the training, participants were encouraged by the course facilitator (LC) to identify creative ways to work together to reduce barriers to help seeking in the Black community. The course facilitator was a member of NHS staff involved in community development, who had experience of providing mental health awareness training and working with marginalised communities. Participants were encouraged to attend each session, to successfully graduate from the course.

### Data analysis

Differences in participants’ scores for each of the secondary outcome measures were calculated and compared at the two time points (*T*_1_–*T*_0_), baseline and one-week post intervention. Tests for normality indicated normal distributions for the MAKS, CAMI, RIBS IB and Disclosure items. Therefore, changes between baseline and post intervention scores for these four measures were analysed using a repeated measures *t* test. Normality tests indicated non-normal distributions for the CAMI Tolerance and Support subscale (CAMI-TS), and the help seeking item, therefore non-parametric Wilcoxon signed-rank tests were performed on the two measures, to compare participants’ scores at baseline and post intervention. Baseline data from participants lost to follow up was not included in the analysis. Descriptive statistics, including means and standard deviations are reported for all outcomes.

For the follow-up assessment, focus groups of participants were held, and audio recorded four weeks following completion of the intervention. Audio recordings were transcribed verbatim, and transcripts analysed using NVivo 12 (QSR [20]). Data were analysed thematically through a series of coding and analysis stages and presented within a thematic framework Braun and Clarke [3]. The analytical process initially involved an inductive approach [3]. The key themes were identified based on the Implementation Science Research Development Tool [17] through a deductive approach. The qualitative findings were validated through cross-checking themes for accuracy with other members of the research team.

## Results

A total of 17 participants were recruited to the study in 2019. All participants completed every session of the ON TRAC intervention. Follow-up data from six participants were not returned, due to the challenges associated with the early stages of the COVID-19 pandemic.

Baseline characteristics for participants’ socio-demographic data are presented in Table 1.

**Table 1:** Participant socio-demographic characteristics (*n* = 17)

	Variable	Count (%)
Age	35–49 50–64 65+	4 (23.5) 10 (58.9) 3 (17.6)
Gender	Female Male Other	11 (64.7) 5 (29.4) 1 (5.9)
Ethnicity	Black African Black Caribbean White British	3 (17.6) 10 (58.8) 4 (23.5)
Religion	Christian	17 (100)
Disability	Yes No Prefer not to say	1 (5.9) 13 (76.5) 3 (17.6)

There were no statistically significant differences between participants’ scores on the MAKS, RIBS, and Disclosure and help-seeking measures, for post intervention compared to baseline. However, a non-significant increase in the mean score on the MAKS, post intervention, suggest that the direction of change in participants’ mental health knowledge was positive. Similarly, the non-significant improvement in mean scores on the RIBS and Disclosure measures suggest a reduction in participants’ desire for social distance, and a greater willingness to disclose personal experiences of mental health problems.

### CAMI—PR

A *t* test found a significant improvement in participants’ mean scores on the CAMI Prejudice subscale, post-intervention (*M* = 11.64) compared to baseline (*M* = 13.45); (*t* (10) = 2.71, *p* = 0.022). The reduction in mean scores indicate a lower level of stigmatising attitudes towards people with mental illness after the intervention (see Table 2).

**Table 2:** Descriptive statistics for outcome measures, collected at baseline and post-intervention (1 week after)

Measure	Pre-intervention (baseline) *n* = 11		Post-intervention *n* = 11	
	Mean	SD	Mean	SD
MAKS	45.73	2.57	47.91	4.97
CAMI—PR	13.45	3.48	11.64	3.38
CAMI—TS	21.73	3.5	24.27	3.41
RIBS	16.55	2.7	17.09	3.11
Disclosure	13.55	3.48	14.18	3.03
Help seeking	17.36	3.36	19.82	3.55

6 participants did not complete post intervention data

### CAMI—TS

A Wilcoxon signed ranks test indicated a significant increase in participants’ scores on the CAMI-TS measure post intervention (Mdn = 24), compared to baseline (Mdn = 22), (*Z* = − 2.037, *p* = 0.042). The improvement in scores suggest an increase in tolerance and support towards people with mental illness after the intervention.

### Willingness test

A Wilcoxon signed ranks test indicated a significant increase in participants scores on the Willingness to Disclose measure post intervention (Mdn = 19), compared to baseline (Mdn = 17), (*Z* = − 2.057, *p* = 0.04). The improvement in scores suggests increased preparedness to seek help amongst participants, less desire for social distance, and greater willingness to engage with people experiencing a mental illness after the intervention.

### Qualitative data

Three key themes, including 9 subthemes were identified from the qualitative data analysis: (i) initial implementation and intention to adopt; (ii) perceived suitability and usefulness of intervention to address cultural issues relating to mental health in the Black community; and (iii) strengthening the capacity of faith leaders.

## Theme 1: initial implementation and intention to adopt.

### Subtheme: implementation activities

Participants (7/7) described intentions to implement mental health activities within their faith groups, following their involvement in the ON TRAC intervention. Faith leaders and faith group members reflected on how they could utilise the resources (trainers, materials), knowledge and skills gained from ON TRAC training course, to raise mental health awareness among their congregation.

The participants (4/7) spoke also about implementing the mental health activities within their own faith groups, which ranged from delivering workshops, seminars and teaching series, to establishing mental health support networks:

### Subtheme: establishing mental health support networks.

Involvement in ON TRAC provided opportunities for participants (6/7) to create a dialogue around mental health issues within their faith groups. Participants (5/7) recognised this as an initial stage in the process of implementing mental health interventions within their faith communities:

For example, the individual mini research project assignment, which involved investigating existing mental health support systems within their faith communities, was instrumental in eliciting interest among faith community members. This resulted in participants identifying other members of their faith communities with an interest in mental health. Participants recognised that there were people within their faith communities with whom they could potentially establish mental health support networks*.*

### Subtheme: engaging key stakeholders

When exploring some of the barriers or challenges associated with discussing mental health within their faith communities, some participants (4/7) highlighted the importance of the involvement of the senior leadership, to support the successful implementation of mental health related activities*.*

One Pastoral Leader experienced some initial resistance from senior leaders within their faith group as a barrier to implementation of activities associated with the ON TRAC intervention. However, he/she noticed that after they had achieved the ‘buy in’ of elders, the process of implementing the intervention activities within their church, was more efficient*.*

Additionally, outreach activities targeted at faith communities, such as short courses delivered by the training facilitator, were identified as key strategies for promoting the intervention to senior faith leaders and faith group members.

## Theme 2: perceived suitability and usefulness of intervention

### Subtheme: increased awareness of stigma

Participants (4/7) described an increase in their awareness of mental health related stigma, and a recognition of the impact of stigma, following their engagement in the intervention*.* Engagement in group discussions and exercises which explored the impact of mental health difficulties, such as case studies and simulation activities, increased self-awareness for participants (4/7). They reported greater awareness of their own unconscious bias and negative attitudes towards mental health and acknowledged the importance of being aware of how these bias impacts on their behaviour, especially when engaging with people who experience mental health difficulties within their faith groups and wider community networks*.*

Along with greater awareness, some participants (4/7) reported a shift in their attitudes towards mental health. However, whilst not all (2/7) thought that their attitudes towards mental health had changed, most spoke (6/7) about developing a better understanding about the ways in which mental health problems can have an adverse impact on different areas of an individual’s life*.*

### Subtheme: greater empathy towards people with mental health needs:

In addition to improving their understanding of the impact of mental health challenges and stigma, many of the participants (6/7) expressed greater emotional empathy and compassion for people experiencing mental health difficulties*.*

Furthermore, when exploring barriers to discussing mental health within faith communities, empathy was highlighted (4/7) as an important factor for overcoming stigma*.*

For one participant, attending the course resulted in increased confidence to address negative stigmatising attitudes and essentially normalise mental health among African people within their faith communities, work, and smaller network of family and friends*.*

### Subtheme: spirituality and mental health

Concerns were expressed about the complexity of the course content, relating to spirituality and mental health. Participants (4/7) highlighted the importance of exploring the relationship between spirituality, pastoral care and mental health. However, the participants (2/7) perceived the information delivered on the course as challenging, ‘*disjointed*’ (Focus group participant 3) and difficult to understand. Participants (3/7) struggled with translating complex *‘psychological’* (Participant 2) models around spirituality and mental health to their physical reality*.*

Given that the participants’ understanding of spirituality derives from the Bible, some (3/7) felt that this topic could have been delivered in a more accessible format, by drawing upon biblical references*.*

There were some conflicting views from participants (3/7) about a lack of structure and order in which the sessions were delivered. Interestingly, one view emerged that it was necessary to explore the topic of mental health within a scientific framework, without reference to Bible scripture. This participant believed that using a medical model to discuss mental health could help broaden faith leaders’ perspectives around mental health and illness and encourage them to consider alternative approaches:

One participant fed back that there was ‘*too much emphasis on the mental health spectrum*’ (Participant 2). Further consideration of the relationship between spirituality, faith and mental health was particularly needed on the course, given that spiritual beliefs have a significant impact on how the Black community respond to people experiencing mental health difficulties or ‘spiritual affliction’. Participants (4/7) reflected on their personal experiences of stigma and discrimination within African churches and their families*.*

## Theme 3: strengthening the capacity of faith leaders.

### Subtheme: improved knowledge and skills

Many of the participants (6/7) perceived the ON TRAC training as an opportunity to build upon previous courses they had completed in mental health, such as Mental Health First Aid, Cognitive Behavioural Therapy (CBT) and Counselling.

Participants reported (4/7) greater knowledge regarding different types of mental health problems and the distinction between mental health and mental illness. They (4/7) also mentioned increased ability and skills in relation to managing emotional distress, human rights and communication.

The human rights component of the training was perceived as particularly relevant given the cultural bias and discrimination that Black people experience in their encounters with health and mental health services. A participant expressed that one of their main motivations for attending the course, was to learn more about how to better engage and support members of the wider Black community, outside of the church. The person stated that by having some knowledge and awareness of human rights, faith leaders could empower Black people to know their rights and make informed choices during their engagement with health services*.*

To demonstrate the significance of human rights training, this participant (1/7) described examples of situations where their friends or family members were potentially detained under the Mental Health Act due to health staff misinterpretation or misunderstanding of the individuals’ behaviour and spiritual experiences.

### Subtheme: improved communication skills and active listening

The Sage and Thyme model [9] delivered on the course was perceived as an effective approach for developing skills in active listening and learning, how to engage with people in an empathic and non-stigmatising way. This model aims to provide clinical and non-clinical staff with evidence-based communication skills to provide person-centred support to someone with emotional concerns Some of the faith leaders mentioned that this was an existing model they were using within their pastoral care. Participants reported (2/7) that this approach helped them learn some key skills for initiating and facilitating conversations with people experiencing distress, in a way that would allow the person to feel respected, validated and understood*.*

### Subtheme: recognising limits and promoting help seeking.

There was an understanding among participants (5/7), to encourage people to seek help from mental health services, if they could not meet the individual’s mental health needs, as faith leaders or faith community members. Participants recognised (3/7) the type of mental health support they thought they could provide, as a result of attending the course and acknowledged that they felt comfortable to support people who may be experiencing mild emotional difficulties. This included: maintaining regular contact with people to reduce isolation; sharing mental health resources; talking about difficulties and emotional care. However, when encountering people with more complex mental health issues group members, said that it is more appropriate to signpost and refer onto NHS services, or the GP as the first point of contact.

In addition, further consultation and two engagement events were held in 2019 to establish how to work effectively with members of local Black faith communities to address issues related to mental health and stigma. Feedback on the course content and format was also collected during these events. They consisted of a ‘Spirituality and mental health training day’, and a consultation day focussed on the ‘ON TRAC Project development with the Spiritual and Pastoral Care in mental health course’. The feedback gained from the events shaped the format and content of the initial version of the ON TRAC training course manual. Following this, the initial version of ON TRAC training course manual was reviewed by the ON TRAC research team. The final version of the initial ON TRAC course manual was created based on all the feedback gathered throughout Phase 2.

## Discussion

The qualitative and quantitative findings suggest that recruitment to the ON TRAC study, delivery of the intervention, conducting quantitative and qualitative assessments and a post group qualitative semi-structured focus group feasible and acceptable to this population. Quantitative results suggest positive changes in mental health knowledge, a reduction in desire for social distance and greater willingness to disclose personal experiences of mental health problems. Qualitative findings for example in subthemes 2.1 and 2.2 further support these findings. The CAMI-PR and TS showed a lower level of stigmatising attitudes and an increase in tolerance and support towards people with mental illness after the intervention. This is further evidenced in subtheme 2.2 where empathy was highlighted as an important factor for overcoming stigma and the increased confidence to address stigmatising attitudes towards mental illness following the intervention. The full attendance of participants to each session further indicates its acceptability to this population.

Faith leaders are often the first point of contact for Black communities when experiencing a crisis (Codjoe et al. [5]), and that people from Black communities are more likely to seek help for mental health problems from faith and community leaders, than health services [18]. The findings from this study tentatively suggest that a manualised intervention to reduce stigma and improve mental health awareness is feasible for Black faith communities. This has implications for further research for example, the development on how spirituality is addressed, and supports the rationale for a future randomised feasibility study.

Building on the scant research in this area, this pilot study also provides some further guidance around the specific elements that are required to create a feasible manualised mental health awareness and stigma reduction intervention in this population and the most helpful mechanisms to achieve this. We can tentatively conclude that the focus on developing relationships, working collaboratively, using a manualised intervention, providing psycho education and skills supported the faith community members to reduce desire for social distance from people with mental health difficulties and show greater willingness to disclose personal experiences of mental health problems. This is in line with findings from a recent extensive review of what is effective for stigma reduction [24]. This noted that the core emerging findings of factors which reduce stigma are in reducing forms of indirect or direct social contact between people who do and who do not have lived experience of mental health conditions, ensuring the intervention content is based on content and culture; close consultation with the identified target group and paying attention to the effects, outcomes and sustainability of the programme.

There are several limitations in this study. As this was a pre-, post-test design, there is a need for a control group in future studies, to test whether the positive changes observed may be due to other reasons such as regression to the mean or other contemporaneous influences on stigma. Missing data may also explain the pre-post changes, although this study was completed under difficult circumstances at the beginning of the first wave of the Covid-19 pandemic. The COVID-19 pandemic began as this study was being completed and raised the possibility that the intervention can be adapted for online delivery. COVID-19 emphasised the importance of addressing mental illness in Black communities and ensure that marginalised and disadvantaged communities can access culturally appropriate and sensitive mental health support during these challenging times [15]. A plan has been implemented to adapt this study for online delivery due to COVID-19 and this later work will be reported in a separate paper.

## Data Availability

Data and materials are available on request.
